# Development of a camelid single-domain antibody-based antigen detection assay for the pan-specific diagnosis of active human and animal *Trypanosoma brucei* infections

**DOI:** 10.1128/jcm.00561-25

**Published:** 2025-10-30

**Authors:** Zeng Li, Bo-kyung Jin, Emily Estefania Timaury Moreno, Andrés Álvarez-Rodríguez, Jo A. Van Ginderachter, Magdalena Radwanska, Yann G. -J. Sterckx, Benoit Stijlemans, Stefan Magez

**Affiliations:** 1 Laboratory of Cellular and Molecular Immunology, Brussels Center for Immunology (BCIM), Department of Bioengineering Sciences (DBIT), Vrije Universiteit Brussel (VUB) 70493, Brussels, Belgium; 2 Department of Biochemistry and Microbiology, Ghent University 366761, Ghent, Belgium; 3 Myeloid Cell Immunology Laboratory, VIB Center for Inflammation Research 206663, Brussels, Belgium; 4 Laboratory for Biomedical Research, Department of Environmental Technology, Food Technology and Molecular Biotechnology, Ghent University Global Campus 549341, Incheon, South Korea; 5 Department of Biomedical Molecular Biology, Ghent University 692902, Ghent, Belgium; 6 Laboratory of Medical Biochemistry (LMB) and the Infla-Med Centre of Excellence, Department of Pharmaceutical Sciences, University of Antwerp 26660, Antwerp, Belgium; Mayo Clinic Minnesota, Rochester, Minnesota, USA

**Keywords:** *Trypanosoma brucei*, single-domain antibody, enolase, diagnosis, sandwich ELISA assay

## Abstract

*Trypanosoma brucei* infections cause African trypanosomiasis, also known as sleeping sickness in humans and nagana in animals, presenting a significant global health and economic burden, especially in sub-Saharan Africa. Animal trypanosomiasis also affects the economic development in Asia and South America. Accurate diagnosis of diseases caused by infection with the parasite of the *T. brucei* group remains a major challenge due to the persistence of infection-induced antibodies long after parasite clearance, complicating serological discrimination between active and past infections. To address this limitation, we developed a sensitive and specific antigen detection assay targeting *Trypanosoma brucei* enolase (*Tbr*ENO) using a panel of camelid single-domain antibodies (sdAbs also known as nanobodies). Among the candidates, the sdAbsR3-77/sdAbR2-103 sandwich enzyme-linked immunosorbent assay (ELISA) exhibited robust performance in detecting circulating *Tbr*ENO in plasma from experimentally infected mice. Additionally, this assay showed strong potential as a “test-of-cure” tool by monitoring real-time antigenemia throughout a chronic *T. brucei* infection course. We further validated the assay’s diagnostic utility in human clinical samples, detecting *Trypanosoma brucei rhodesiense* infections at both early and advanced stages and *Trypanosoma brucei gambiense* infections in advanced stage. The sdAbsR3-77H/sdAbR2-103HA ELISA targeting *Tbr*ENO shows potential for point-of-care pan-diagnosis of active *T. brucei* infections (including *Trypanosoma brucei brucei*, *T. b. gambiense*, *T. b. rhodesiense*, *Trypanosoma brucei evansi*, and *Trypanosoma brucei equiperdum*) in both animals and humans. Therefore, this assay addresses gaps in current diagnostic capabilities by overcoming the key limitations of antibody-based tests, offering a promising tool for improved disease control.

**IMPORTANCE:**

African trypanosomiasis, commonly known as sleeping sickness in humans and nagana in animals, is a life-threatening disease that remains a major health and economic concern in many parts of the world. One of the key difficulties in managing this disease is detecting ongoing infections, as existing antibody-based tests cannot reliably distinguish between current and past infections. In this study, we developed a novel laboratory test that detects a specific protein released by the parasite during infection. This test uses special antibodies derived from camels, known for their exceptional stability and precision, to accurately identify infections caused by multiple *Trypanosoma brucei* subspecies. Our approach not only enables accurate diagnosis but also offers a way to monitor treatment success. This work provides a valuable tool for disease control efforts and could help improve the health of both humans and animals in regions where trypanosomiasis is endemic.

## INTRODUCTION

*Trypanosoma brucei* is an extracellular protozoan parasite causing African trypanosomiasis (AT) in humans and animals (human African trypanosomiasis [HAT] and animal African trypanosomiasis [AAT], respectively). *T. brucei* encompasses the subspecies *Trypanosoma brucei gambiense*, *Trypanosoma brucei rhodesiense*, *Trypanosoma brucei brucei*, *Trypanosoma brucei evansi*, and *Trypanosoma brucei equiperdum*, with the first two causing HAT (1, 2). Notably, *T. b. rhodesiense* is responsible not only for HAT but also for infecting a wide range of wildlife species and domestic cattle, making it a causative agent of both HAT and AAT (3). HAT is a severe, life-threatening disease that leads to neurological deterioration, including symptoms such as drowsiness, cognitive changes, and coma. If left untreated, particularly in chronic cases of *T. b. gambiense*, HAT is considered to most often progress to a fatal stage, as is the case for *T. b. rhodesiense*. In contrast, AAT primarily affects livestock, presenting with symptoms such as fever, anemia, and weight loss, but does not directly pose a threat to human life, with the exception of the zoonotic transmission of *T. b. rhodesiense*. While AAT can result in significant economic losses, its clinical impact is generally less severe than HAT, as it does not present the same neurological damage as seen in humans (4). Although *T. b. evansi* is classified as a veterinary parasite, zoonotic events have been documented (5, 6). Currently, there is no available prophylactic vaccine for AT, and control relies on early diagnosis and treatment of infected individuals, as well as vector management. While treatment would ideally be initiated only after confirmation of active parasitemia, rapid diagnostic tests available to date can only score for exposure, not infection (7, 8). Therefore, the development of a diagnostic test for infection is paramount to support AT control strategies.

Visualization of *T. brucei* trypomastigotes by microscopy is the “gold standard” for AT diagnosis, but this technique has low sensitivity and is problematic during both early and chronic infection stages (9, 10). Nucleic acid-based diagnostic assays for AT have higher sensitivity and/or specificity but are unsuitable as point-of-care (POC) technology due to their high equipment and reagent demand (11–14). Serology-based diagnostic tests, such as antibody (Ab) detecting enzyme-linked immunosorbent assays (ELISAs) or lateral flow devices derived thereof, rely on the host’s immune response for signal amplification. However, disadvantages include (i) low sensitivity during early infection stages when Ab levels fall below the detection limit; (ii) reduced specificity due to infection-triggered cross-reactivity; and (iii) persistent Abs remaining long after infection clearance, resulting in false-positive test outcomes due to the inability to distinguish between active and past infections (15, 16). These issues can be overcome using antigen-based immunoassays. Antigen-based tests offer several significant advantages: (i) they enable direct detection of the presence of parasite proteins, providing definitive confirmation of active infection rather than relying on host immune responses, which may only indicate past exposure; (ii) they offer enhanced diagnostic accuracy by targeting parasite-specific antigens, reducing the chances of false positives, hence improving positive predictive value (PPV), which in itself becomes the most crucial parameter when working in a low-prevalence setting; (iii) they prompt treatment decisions by delivering rapid and accurate results, preventing the disease progression to more severe stages; and (iv) they are typically designed as rapid diagnostic tests, which are user-friendly and require minimal training, making them ideal for rural clinics or mobile health units. Despite previous efforts to develop antigen-based tests for *T. brucei* using monoclonal antibodies, the critical process of validating these tests has largely been overlooked (17, 18). Finally, it is important to note here that Ag-based tests that rely on the use of conventional antibodies (monoclonal or polyclonal) are inherently susceptible to molecular competition between the antibodies included in the manufacturer’s test kit and those produced by the host in response to the infection. Notably, the host’s antibodies may be of higher quality as they would be raised against the exact epitopes presented by the pathogen to the immune system. A bottleneck here is that the Ab pairs employed in antigen-based immunoassays should be able to outcompete infection-induced host Abs (19, 20).

Camelid single-domain antibodies (sdAbs also known as nanobodies) hold great promise to overcome the hurdles encountered with conventional Abs used in antigen-based immunoassays (21). sdAbs correspond to the variable antigen binding domain of a heavy-chain-only Ab subset found in camelids (22, 23). To compensate for the loss of the light chain, sdAbs display an increased solubility and longer complementarity-determining regions (CDRs, especially CDR3), thereby maintaining a large enough paratope surface area for antigen recognition. Hence, sdAbs possess unique paratope features, which enable them to recognize unique and cryptic antigenic sites that are typically not accessible to conventional Abs (24). In addition, sdAbs display very little to no cross-reactivity with host Abs (25). Along with their high thermostability, solubility, and facile recombinant production in bacteria or yeast, sdAbs possess numerous advantages for the development of diagnostic and therapeutic applications.

Here, we describe a pan-specific antigen-based immunoassay for the detection of active *T. brucei* infections by targeting *Trypanosoma brucei* enolase (*Tbr*ENO) through camelid sdAbs. *Tbr*ENO (also known as *Tb/ev*ENO or *Tev*ENO) was recently discovered as a potential biomarker for the sdAb-based detection of both *T. b. evansi* type A and type B infections (26). Given that *Tbr*ENO is 100% conserved among all subspecies, it emerges as a promising candidate for the pan-specific detection of *T. brucei* infections. In this article, we demonstrate that the sdAbsR3-77H/sdAbR2-103HA sandwich ELISA assay is capable of detecting *Tbr*ENO in plasma samples from experimentally infected mice and show its use as a “test-of-cure” tool. Finally, we demonstrate ELISA’s potential for correctly scoring HAT samples, paving the road toward the development of a *Tbr*ENO-based POC diagnosis of both human and animal *T. brucei* infections.

## MATERIALS AND METHODS

### Production and purification of recombinant proteins

#### Production and purification of sdAbs

The sdAb genes cloned into the pMECs vector were amplified by PCR followed by double digestion with *Pst*I and *Eco*91I and ligation into pHen6c vector for constructing of a hex histidine-tag. The pHen6c constructed sdAb gene was transformed in *Escherichia coli* WK6 cells for expression, and periplasmic (PE) extract was obtained as previously described (26). The PE extracts containing sdAbs were then incubated with His-Select Nickel resin (Sigma) for 1 h at room temperature (RT) and subsequently were purified as described previously by us using a standard protocol with size exclusion chromatography (SEC) through a Superdex 75 16/60 column (GE Healthcare) and stored at 4°C (26).

#### Production and purification of recombinant *T. brucei*, *Trypanosoma congolense*, and *Trypanosoma vivax* enolase

The recombinant production and purification of *Tbr*ENO was previously described by Li et al. (26). The gene encoding *Trypanosoma congolense* enolase (*Tco*ENO) (TriTrypDB ID: TclL3000.A.H_000739900) and *T. vivax* enolase (*Tvi*ENO) (TriTrypDB ID: TvY486_1002910) were codon optimized for expression in *E. coli*. Recombinant production and purification of *Tco*ENO and *Tvi*ENO were as described for *Tbr*ENO, and aliquots were stored at −20°C.

After thawing on ice, aliquots were lysed by sonicating at 20% amplitude for 90 sonication cycles of 5 s pulse with 5 s pause between each cycle (Bioblock Scientific Vibro Cell 75043), and lysates were centrifuged for 30 min at 30,966 × *g*, 10°C. Supernatants were then passed through a filter with a pore size of 0.45 µm. The recombinant *Tbr*ENO, *Tco*ENO, and *Tvi*ENO proteins were purified by IMAC and SEC using an NGC Chromatography System (Bio-Rad) as described by us previously (26). Each purification step was monitored by SDS-PAGE under reducing conditions, followed by staining with Coomassie blue.

### ELISA

Nunc MaxiSorp flat-bottom 96-well plates (Thermo Fisher Scientific) were used in a standard ELISA protocol, in which the following incubation steps were performed: (i) capture sdAbs diluted in 100 µL coating buffer (0.1 M NaHCO_3_, pH 8.2); (ii) blocking with 300 µL SuperBlock T20 (phosphate-buffered saline [PBS]); (iii) detection sdAbs diluted in 100 µL blocking buffer; (iv) 100 µL of antihemagglutinin (HA)-tagged biotinylated antibody (Thermo Fisher Scientific) diluted 1:4,000 in blocking buffer; (v) 100 µL of streptavidin-HRP (BioLegend) diluted 1:4,000 in blocking buffer; (vi) 100 µL of 3,3′,5,5′-tetramethylbenzidine substrate (Sigma); and (vii) the addition of 50 µL 1 M H_2_SO_4_. After 10 min for assay development, optical density at 450 nm was determined using a Varioskan LUX multimode microplate reader (Thermo Fisher Scientific).

For experiments involving native *Tbr*ENO or heparin-prepared plasma samples from mice, 96-well half-area microplates (Corning) were used with 1/2 volumes.

#### Sandwich ELISA combination assay

The five sdAb candidates were tested for their ability to form a *Tbr*ENO sandwich ELISA. His (H)-tagged capturing sdAbs (sdAb11H, sdAbR1-10H, sdAbR1-45H, sdAbR2-103H, and sdAbsR3-77H) were coated at 4°C overnight at a concentration of 2 µg/mL. Recombinant *Tbr*ENO at a final concentration of 2 µg/mL was added to each coated well at a volume of 100 µL. HA-tagged detecting sdAbs (sdAb11HA, sdAbR1-10HA, sdAbR1-45HA, sdAbR2-103HA, and sdAbsR3-77HA) were added at a concentration of 2 µg/mL. The rest of the ELISA is as described above.

The specificity of sdAb11H/sdAbR1-10HA and sdAbsR3-77/sdAbR2-103 sandwich ELISA assays was determined on recombinant *Tbr*ENO, *Tco*ENO, and *Tvi*ENO using the abovementioned ELISA setup. The concentration used in the sdAb11/sdAbR1-10 sandwich ELISA assay was 10 µg/mL for both capturing and detecting sdAbs, while the concentration used for the sdAbsR3-77/sdAbR2-103 pair was 5 µg/mL for both sdAbs.

The sensitivity of the two sdAb pairs was determined on recombinant *Tbr*ENO in a twofold serial dilution starting from 2 µg/mL, whereas the sensitivity of sdAbsR3-77H/sdAbR2-103HA was also assessed using plasma from *T. brucei*-infected mice. The concentrations of the sdAbs and the ELISA setup were the same as mentioned above.

#### Checkerboard titration of sdAb pairs sdAb11H/sdAbR1-10HA and sdAbsR3-77H/sdAbR2-103HA

Optimal concentrations for sdAb pairs were determined through checkerboard titrations. Briefly, a twofold serial dilution of capturing sdAbs (sdAb11H and sdAbsR3-77H) starting from 10 µg/mL was coated overnight in replicates. Recombinant *Tbr*ENO was added to each coated well at a concentration of 2 µg/mL. Detecting sdAbs (sdAbR1-10HA and sdAbR2-103HA) was titrated in the same way as the capturing sdAbs. The ELISA setup was the same as mentioned above.

#### Assessing the possibility of a plasma matrix effect on the sdAb11H/sdAbR1-10HA and the sdAbsR3-77H/sdAbR2-103HA sandwich ELISA assays

To verify the plasma matrix effect for the two sdAb pair sandwich ELISA assays, *Tbr*ENO spiked in mouse plasma was used. The concentrations of the sdAbs and the ELISA setup were the same as mentioned above.

### Flow cytometry assay

Purified, fixed, and permeabilized *T. brucei* (AnTat1.1E) parasites were tested for recognition of *Tbr*ENO by the anti-*Tbr*ENO sdAbs. *T. brucei* (AnTat1.1E) parasites were diethylaminoethyl (DEAE)-purified from infected mouse blood (27), washed three times in phosphate saline glucose buffer (pH 8) through centrifugation (805 × *g* for 10 min at 4°C), and aliquoted at 2 × 10^7^ parasites/mL. Parasites were fixed and permeabilized by gently resuspending cells in 200 µL of BD Cytofix/Cytoperm (Thermo Fisher Scientific) and followed by a 20 min incubation at 4°C. After washing with BD Perm/Wash buffer (Thermo Fisher Scientific) (1 mL/tube), parasites were resuspended in 1 mL Perm/Wash buffer. Next, 100 µL of parasite suspension was incubated for 30 min at 4°C with 5 µg of each HA-tagged sdAb, washed with 200 µL Perm/Wash buffer, and incubated with 1 µg of an Alexa Fluor 488-conjugated anti-HA IgG (BioLegend) (1/1,000 dilution in the Perm/Wash buffer) for 30 min at 4°C. After a final washing with 200 µL Perm/Wash buffer, parasites were resuspended in 150 µL washing buffer for FACSCanto II flow cytometer (BD) data recording. Data were analyzed using FlowJo software 10.

### Comparison between sdAbsR3-77H/sdAbR2-103HA sandwich ELISA and microscopy in an experimental mouse infection model

C57BL/6J mice (8 weeks old, Janvier) were inoculated intraperitoneally with 5,000 *T. brucei* (AnTat1.1E) parasites and treated with melarsoprol (10–12 mg/kg), provided by the World Health Organization [WHO]/Sanofi) at days 7–10 post-infection (28). The enolase plasma antigenemia was evaluated using the sdAbsR3-77H/sdAbR2-103HA sandwich ELISA assay and microscopy on different days post-infection by tail vein bleed (100 µL). Plasma was obtained by centrifugation at 20,817 × *g* for 10 min and diluted 1/4 in SuperBlock for ELISA testing.

### Mouse plasma samples

Plasma samples from mice infected with *Plasmodium chabaudi* and *Leishmania infantum* were obtained from Philippe Van den Steen (KU Leuven) and Guy Caljon (UAntwerpen), respectively. For each pathogen, plasma was collected from four individual animals at peak parasitemia and confirmed positive using a thin Giemsa-stained smear for *P. chabaudi* and bioluminescence imaging for *L. infantum*. Plasmas from four *T. brucei*-infected mice at peak parasitemia, which were confirmed by microscopy, and from naïve mice were used as positive and negative controls, respectively. All samples were used to validate the specificity of the sdAbsR3-77H/sdAbR2-103HA sandwich ELISA.

### Preparation of parasite lysate

*T. brucei* parasites were isolated from the blood of infected mice using a DEAE-52 column as described before (ref. 27). Parasites were cultured in HMI9 medium plus 20% fetal calf serum (Gibco). After two days of *in vitro* culture, parasites were collected following centrifugation (805 × *g*, 10 min, RT). *Trypanosoma cruzi* parasites were obtained from *in vitro* cultures from Domagalska Malgorzata (ITM). *T. brucei* and *T. cruzi* soluble lysate proteins were prepared by resuspending 10^8^ parasites of each species in 200 µL of PBS, followed by sonication for four cycles of 15 s pulses with a 15 s pause between each cycle while on ice. Following centrifugation (20,817 × *g*, 10 min, 4°C), the soluble protein fraction was collected, and the protein concentrations were determined using a NanoDrop. For ELISA, 100 ng of each soluble lysate was used in the sandwich system as described in the section ELISA.

### Human plasma samples

Human plasma samples were obtained from the WHO/HAT/Specimen Bank, including 20 confirmed *T. b. rhodesiense* and *T. b. gambiense* HAT cases, evenly distributed between the two stages of infection. Ten negative control samples were also provided. All samples were used to validate the sdAbsR3-77H/sdAbR2-103HA sandwich ELISA.

### Statistical analysis

The GraphPad Prism 10 software was used for statistical analyses. A one-way analysis of variance (ANOVA) with Kruskal-Wallis test with multiple comparisons, an ordinary one-way ANOVA with multiple comparisons, or unpaired *t*-tests with Mann-Whitney test were performed. Values are expressed as mean ± SD or median, where *P* ≤ 0.05 is considered statistically significant.

## RESULTS

### Two sdAb pairs efficiently detect *Tbr*ENO in a sandwich ELISA format

**Fig 1:**
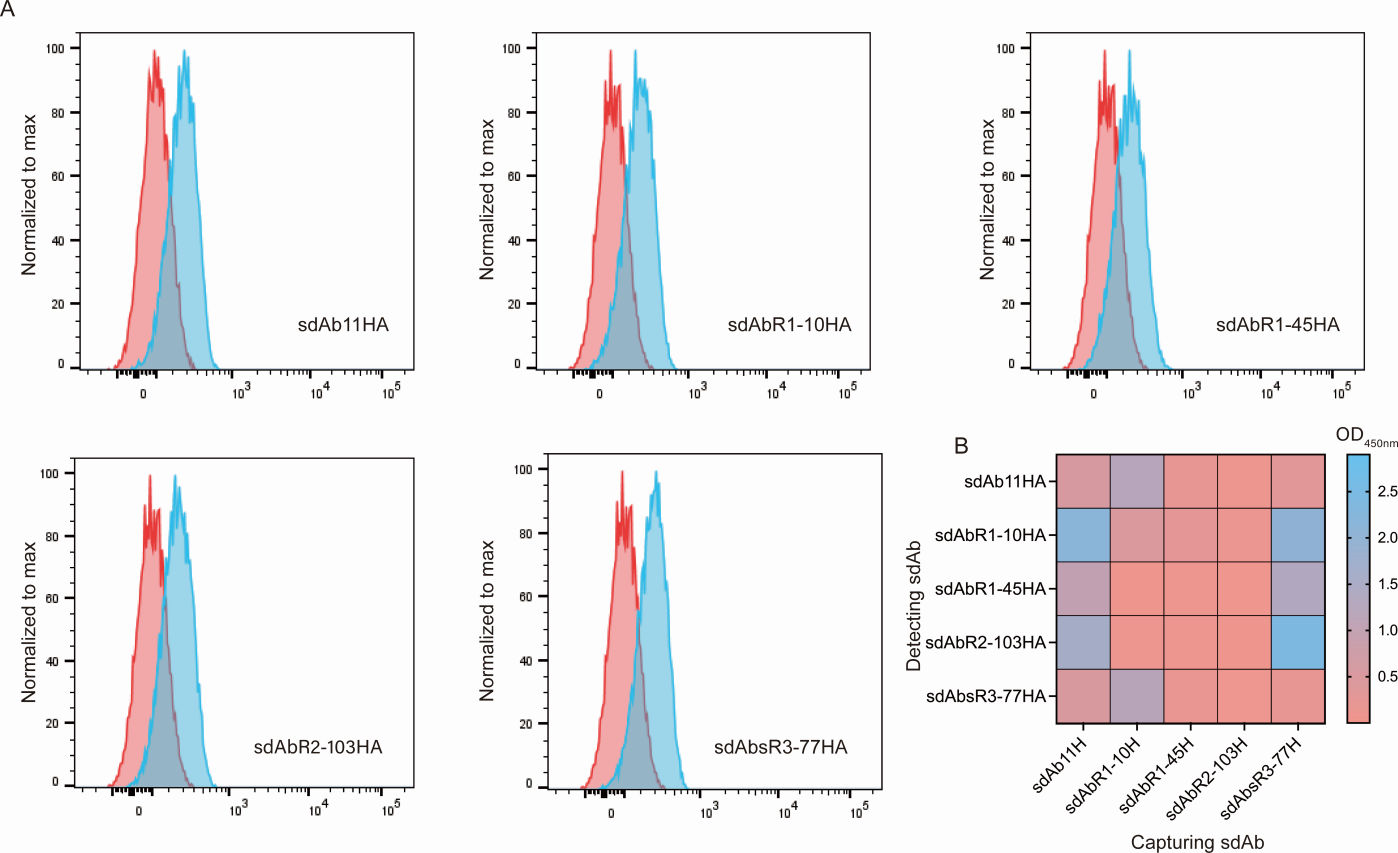
Anti-*Tbr*ENO sdAbs recognize their native antigen on fixed and permeabilized parasites via flow cytometry, and two sdAb pairs efficiently detect *Tbr*ENO in a sandwich ELISA format. (**A**) Flow cytometry analysis of five anti-*Tbr*ENO sdAbs binding to purified, fixed, and permeabilized *T. brucei* (AnTat1.1E) parasites. Signals of parasites in the presence of Alexa Fluor 488-labeled anti-hemagglutinin (HA) IgG alone (red) and parasites in the presence of sdAbs and Alexa Fluor 488-labeled anti-HA IgG (blue) are shown. (**B**) Heatmap representation of the results of the sdAb sandwich pairing assay performed with five anti-*Tbr*ENO sdAbs (sdAb11, sdAbR1-10, sdAbR1-45, sdAbR2-103, and sdAbsR3-77). In the setup, His (H)-tagged and HA-tagged sdAbs were both deployed as capturing and detecting sdAb, respectively. Results are representative of two independent experiments, and each condition consists of technical duplicates (*n* = 2).

Five sdAbs (sdAb11, sdAbR1-10, sdAbR1-45, sdAbR2-103, and sdAbsR3-77), previously identified as *Tbr*ENO binders (26), were tested in flow cytometry on purified, fixed, and permeabilized *T. brucei* parasites (Fig. 1A). Higher fluorescent signals could be observed for all sdAbs, indicating efficient recognition of native *Tbr*ENO. Next, the most performing sdAb pairs were identified by testing 25 sandwich ELISA combinations. In this setup, H- and HA-tagged sdAbs were used as capturing and detecting molecules. Eight sdAb pairs (sdAb11H/sdAbR1-10HA, sdAb11H/sdAbR1-45HA, sdAb11H/sdAbR2-103HA, sdAbR1-10H/sdAb11HA, sdAbR1-10H/sdAbsR3-77HA, sdAbsR3-77H/sdAbR1-10HA, sdAbsR3-77H/sdAbR1-45HA, and sdAbsR3-77H/sdAbR2-103HA) gave a detectable signal (Fig. 1B), with sdAb11H/sdAbR1-10HA and sdAbsR3-77H/sdAbR2-103HA generating the highest signals. Here, the sdAb detecting and capturing roles are non-interchangeable, as combinations of the sdAbR1-10H/sdAb11HA and sdAbR2-103H/sdAbsR3-77HA pairs failed to yield comparable signals. Consequently, the sdAb11H/sdAbR1-10HA and sdAbsR3-77H/sdAbR2-103HA pairs were selected for further development.

### The identified sdAb pairs specifically recognize *Tbr*ENO in simple and complex matrices

**Fig 2:**
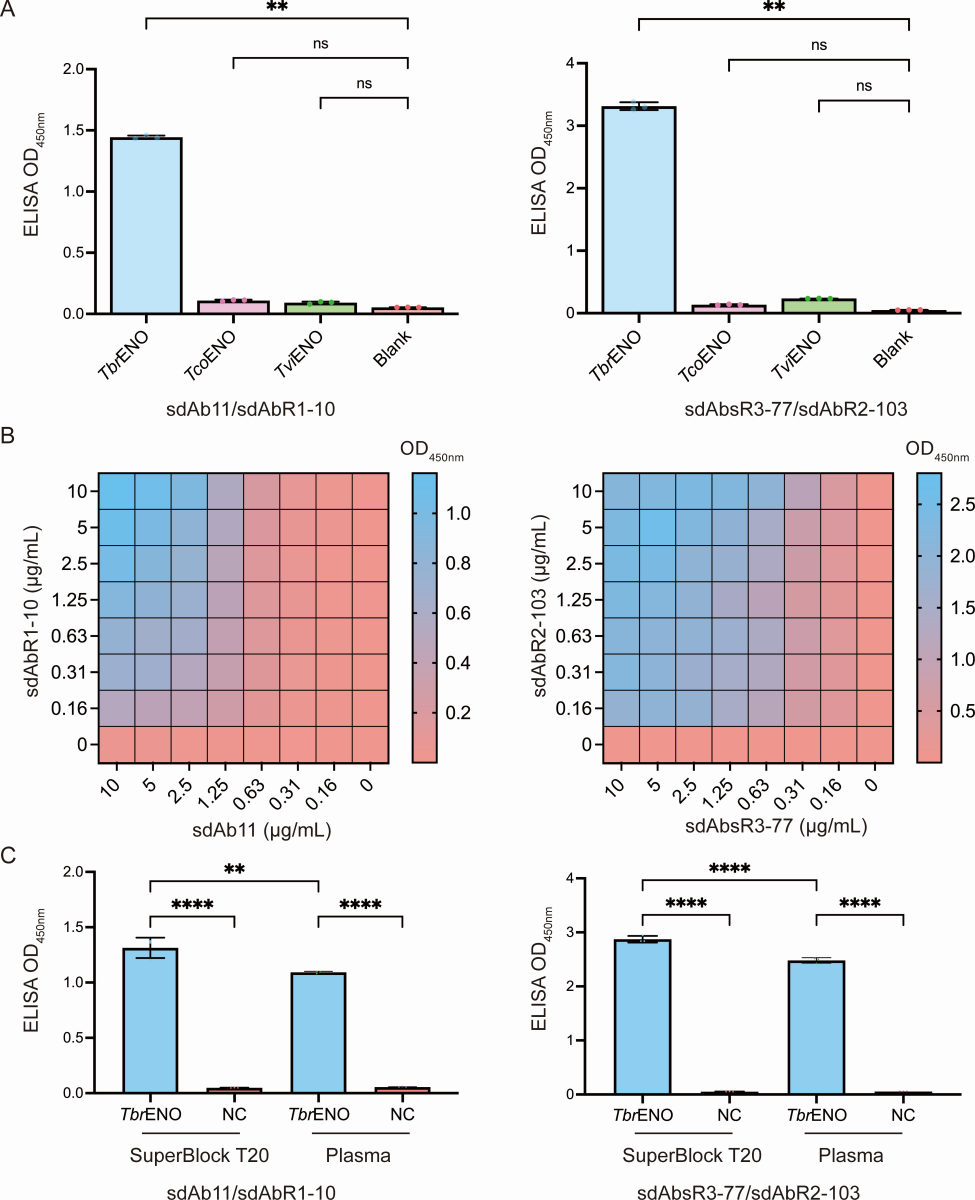
Optimization of the *Tbr*ENO-specific Nb-based sandwich ELISA assays to overcome the plasma matrix effect. (**A**) sdAb11H/sdAbR1-10HA and sdAbsR3-77H/sdAbR2-103HA sandwich ELISA assays on recombinant *Tbr*ENO, *Tco*ENO, and *Tvi*ENO demonstrate their specificity only for *Tbr*ENO. A one-way ANOVA (Kruskal-Wallis test with multiple comparisons) was performed, whereby each sample was compared to the blank. Results are representative of two independent experiments (*n*  =  3) and presented as mean ± SD. **P ≤ 0.01. ns, not significant. (**B**) Heatmaps readily identify the optimal practical setup to conduct the sdAb11H/sdAbR1-10HA and sdAbsR3-77H/sdAbR2-103HA sandwich ELISA assays. To identify the optimal conditions yielding the highest signals, a checkerboard ELISA with varying amounts of capturing and detecting sdAbs was assessed. (**C**) Investigation of the potential plasma matrix effect for sdAb11H/sdAbR1-10HA and sdAbsR3-77H/sdAbR2-103HA sandwich ELISA assays. Naïve mouse plasma and SuperBlock T20 blocking buffer were spiked with recombinant *Tbr*ENO at a concentration of 1 µg/mL. An ordinary one-way ANOVA with multiple comparisons was performed. Results are representative of two independent experiments (*n* = 3) and presented as mean ± SD. **P ≤ 0.01, ****P ≤ 0.0001. Non-significant differences are not shown.

To evaluate specificity, ELISA assays were performed with recombinant proteins from *T. brucei*, *T. congolense*, and *T. vivax* (*Tbr*ENO, *Tco*ENO, and *Tvi*ENO). Figure 2A shows strict specificity for *Tbr*ENO, with no detection of *Tco*ENO or *Tvi*ENO. A checkerboard analysis determined that for the sdAb11H/sdAbR1-10HA pair, the highest ELISA signal was observed when 10 µg/mL of both capturing and detecting sdAb were employed (Fig. 2B, left panel), while for the sdAbsR377H/sdAbR2-103HA pair, the highest ELISA signal was obtained when both capturing and detecting sdAb were used at 5 µg/mL (Fig. 2B, right panel).

Next, the plasma matrix effect was assessed, which could allow target antigen detection in biological samples (28). Therefore, recombinant *Tbr*ENO-spiked naïve mouse plasma and SuperBlock T20 blocking buffer were used. As shown in Fig. 2C, a minimal, yet significantly lower signal was obtained when using mouse plasma compared to using the SuperBlock T20 blocking buffer. However, the plasma matrix effect was not as drastic as previously reported (28), given that the overall detection signal (value) in the two different buffers was in the same range. Interestingly, the ELISA signals observed for the sdAbsR3-77H/sdAbR2-103HA pair are consistently higher than those for the sdAb11H/sdAbR1-10HA pair, suggesting that the sdAbsR3-77H/sdAbR2-103HA pair exhibits greater sensitivity.

### The sdAbsR3-77H/sdAbR2-103HA pair exhibits greater sensitivity compared to the sdAb11H/sdAbR1-10HA pair

**Fig 3:**
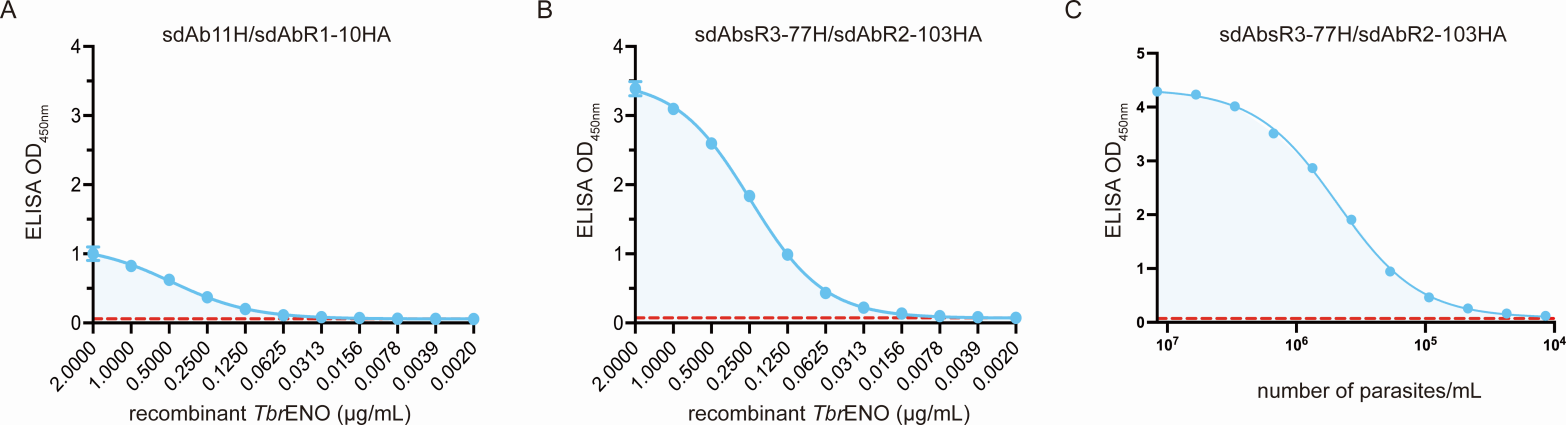
Limit of detection (LoD) of *Tbr*ENO-specific sandwich ELISA assays. LoD of sdAb11H/sdAbR1-10HA (**A**) and sdAbsR3-77H/sdAbR2-103HA (**B**) against recombinant *Tbr*ENO. (**C**) LoD of sdAbsR3-77H/sdAbR2-103HA against plasma from *T. brucei*-infected mice (day 7 post-infection), detecting the antigen which is correlated to the parasitemia (microscopically). The dashed line (red) displays the ELISA LoD (LoD = mean blank + 2 × SD_blank_). Results are representative of two independent experiments, and each condition consists of technical triplicates (*n* = 3).

The limit of detection (LoD) of both promising sdAb sandwich ELISAs was determined using a twofold serial dilution of recombinant *Tbr*ENO, starting from 2 µg/mL (Fig. 3, panels A and B). While the sdAb11H/sdAbR1-10HA pair displays a LoD of 125 ng/mL (Fig. 3A), the LoD observed for the sdAbsR3-77H/sdAbR2-103HA pair under the same experimental conditions is around fourfold lower (32 ng/mL, Fig. 3B), confirming that the sdAbsR3-77H/sdAbR2-103HA combination exhibits a higher sensitivity. Next, plasma was isolated from mouse whole blood at peak parasitemia (1.2 × 10^8^ parasites/mL) and subsequently subjected to a twofold dilution across 11 dilutions. As depicted in Fig. 3C, the sdAbsR3-77H/sdAbR2-103HA pair can effectively detect approximately 50,000 T. *brucei* parasites/mL.

### The sdAbsR3-77H/sdAbR2-103HA pair allows specific detection of active *T. brucei* infections in an experimental mouse model

Next, the sdAbsR3-77H/sdAbR2-103HA sandwich ELISA was assessed for its ability to distinguish between active and past infections in an experimental mouse model. In this experiment, C57BL/6J mice were divided into four groups, with five individuals in each group. The correlation between antigenemia (detected via the sdAbsR3-77H/sdAbR2-103HA sandwich ELISA) and parasitemia (observed via light microscopy) was determined by analyzing blood samples from mice infected with *T. brucei* AnTat1.1 parasites (groups 1 and 2) and uninfected control mice (groups 3 and 4) at various time points post-infection. In addition, groups 1 and 3 were left untreated, while groups 2 and 4 were treated with melarsoprol for four consecutive days starting at day 7 post-infection.

**Fig 4:**
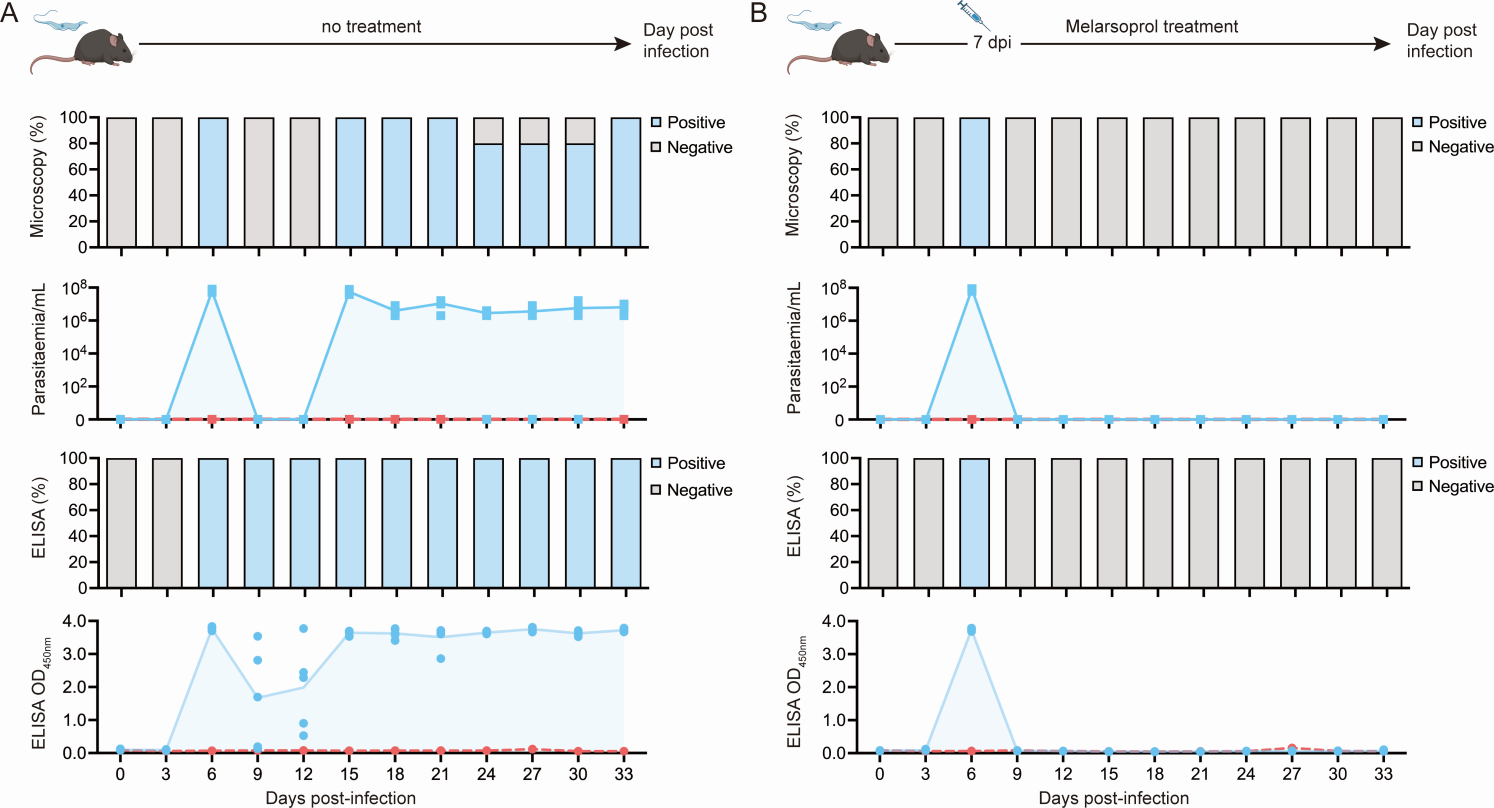
Evaluation of the sdAbsR3-77H/sdAbR2-103HA sandwich ELISA assay as a test-of-cure tool in a *T. brucei*-infected mouse model. (**A**) C57BL/6J mice (*n* = 5) were infected with *T. brucei* (AnTat1.1) parasites. Antigenemia and parasitemia were monitored over the course of the infection by microscopy (top two panels) and the sdAbsR3-77H/sdAbR2-103HA sandwich ELISA (bottom two panels), respectively. The results are displayed as the percentages of mice that scored positive or negative, and the mean values for parasitemia and antigenemia are shown as a blue line and light blue fill, respectively. The dashed line (red) displays the naïve mice and ELISA detection cut-off value. (**B**) C57BL/6J mice (*n* = 5) were infected with *T. brucei* (AnTat1.1) parasites and treated with melarsoprol at 7 days post-infection. The presence of parasites was analyzed by microscopy (top two panels) and the sdAbsR3-77H/sdAbR2-103HA sandwich ELISA assay (bottom two panels) throughout the experiment. The panels and color codes are the same as for panel A.

The sdAbsR3-77H/sdAbR2-103HA sandwich ELISA and microscopy results correlate for most of the collected samples (Fig. 4A), with both antigenemia and parasitemia being detected starting at day 6 post-infection. A significant discrepancy between the two detection methods was observed at days 9 and 12 post-infection, where parasites could not be detected in any of the mice by microscopy, while all samples were positive when examined using the sdAbsR3-77H/sdAbR2-103HA sandwich ELISA. Additionally, on days 24, 27, and 30 post-infection, parasites could not be detected by microscopy in one out of five mice, but all samples remained positive when tested with the sdAbsR3-77H/sdAbR2-103HA sandwich ELISA until the end of the infection (day 33 post-infection). These results show the superior sensitivity of the sdAb ELISA compared to parasite detection by light microscopy. Interestingly, *Tbr*ENO signals become undetectable within 48 h after melarsoprol treatment, indicating that parasite clearance coincides with the quick disappearance of the target antigen from the host bloodstream (Fig. 4B). This demonstrates that the sdAbsR3-77H/sdAbR2-103HA *Tbr*ENO-specific sandwich ELISA detects only active *T. brucei* infections and would thus qualify as a suitable test-of-cure tool.

### The sdAbsR3-77H/sdAbR2-103HA sandwich assay can detect *T. b. rhodesiense* and *T. b. gambiense* infections in human plasma

**Fig 5:**
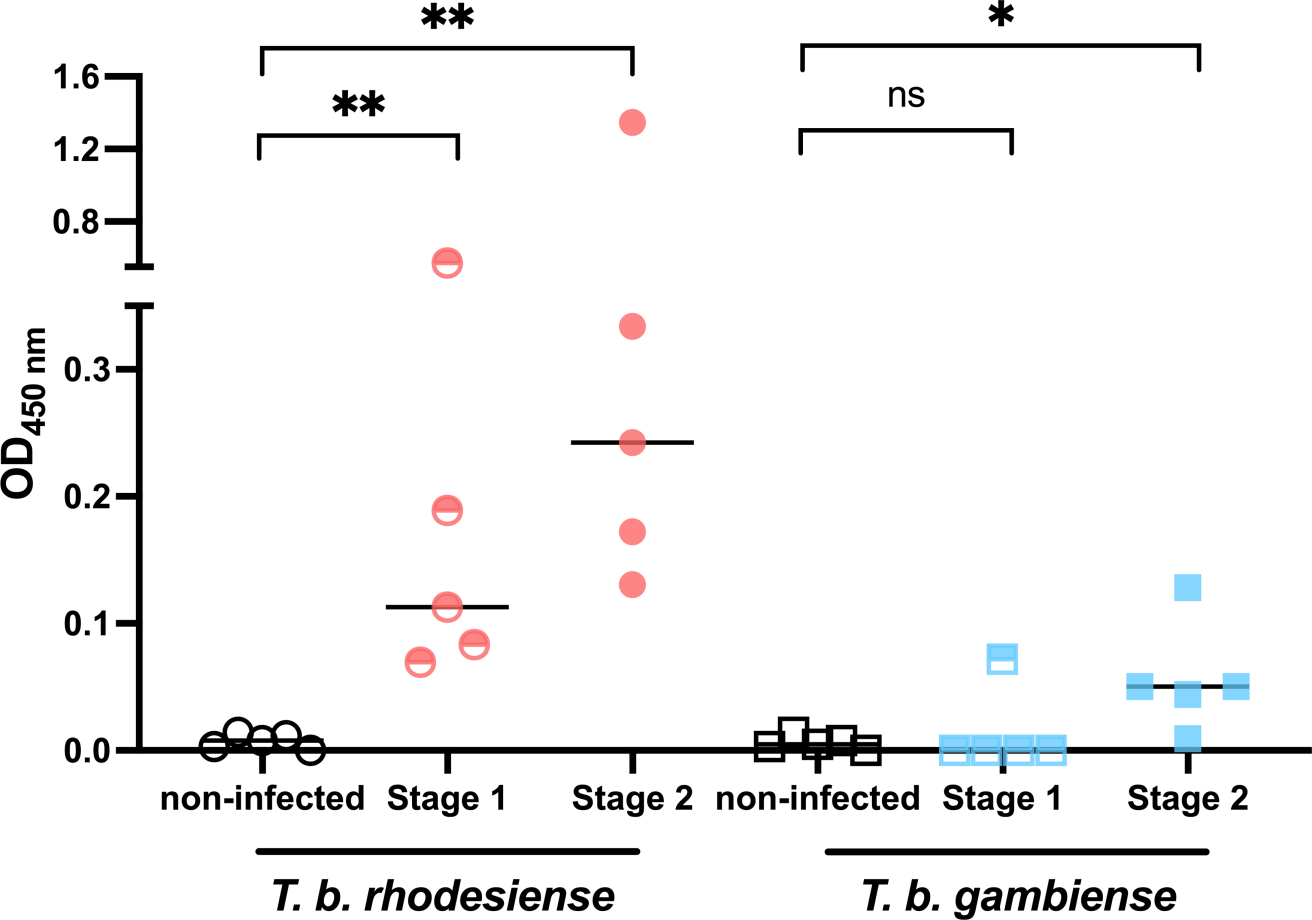
Validation of the sdAbsR3-77H/sdAbR2-103HA sandwich ELISA for detecting human *T. b. rhodesiense* and *T. b. gambiense* infections in plasma. For each group, five samples were used, and the result is presented as median. The optical density at 450 nm (OD_450nm_) signal was calculated by subtracting the background signal measured from wells that did not contain the target antigen. An unpaired *t*-test with Mann-Whitney test was performed. Results are presented as median. *P ≤ 0.01, **P ≤ 0.001. ns, not significant.

To evaluate the effectiveness of the sdAbsR3-77H/sdAbR2-103HA sandwich ELISA for detecting HAT, the test was preliminarily validated using 30 individual plasma samples obtained from the WHO/HAT/Specimen Bank. These samples were obtained from 20 individuals with confirmed *T. b. rhodesiense* or *T. b. gambiense* infections, comprising 10 first-stage cases and 10 second-stage cases. Ten samples from uninfected individuals were provided as negative controls. The results in Fig. 5 show that all 10 samples derived from *T. b. rhodesiense*-infected individuals scored above the negative control samples using the sdAbsR3-77H/sdAbR2-103HA sandwich ELISA. Interestingly, the signals obtained in late-stage patients were higher than those observed for acute stage patients. In contrast, for samples derived from patients infected with *T. b. gambiense*, only one first-stage *T. b. gambiense* case was positive, while four out of five second-stage samples scored positive. The test did not yield any false-positive results among the 10 negative control samples, indicating no cross-reactivity with healthy/non-infected human individuals. Therefore, the results suggest that the sdAbsR3-77H/sdAbR2-103HA sandwich ELISA has the potential to serve as an effective tool for pan-specific detection of *T. b. rhodesiense* HAT infections at both infection stages, as well as *T. b. gambiense* HAT infections in the second stage.

## DISCUSSION

Several antigens have been proposed as biomarkers for the diagnosis of *T. brucei* infections (38–40), including three trypanosomal glycolytic enzymes identified by us: *T. congolense* fructose-1,6-bisphosphate aldolase, *T. congolense* pyruvate kinase, and *Tbr*ENO (25, 26, 29). Here, the potential use of the latter was investigated using five anti-*Tbr*ENO sdAbs (sdAb11, sdAbR1-10, sdAbR1-45, sdAbR2-103, and sdAbsR3-77). Given that *Tbr*ENO is 100% identical at the amino acid level for all *T. brucei* subspecies, we were able to identify two pan-reactive sdAb pairs (sdAb11H/sdAbR1-10HA and sdAbsR3-77H/sdAbR2-103HA) that do not cross-react with *Tco*ENO and *Tvi*ENO. This is remarkable, given that *Tco*ENO and *Tvi*ENO share ~90% and ~88% sequencing identity with *Tbr*ENO at the amino acid level, respectively (see Fig. S1A).

In an ELISA format, the highest signals are observed at concentrations of 10 and 5 µg/mL for both sdAbs in the sdAb11H/sdAbR1-10HA and sdAbsR3-77H/sdAbR2-103HA pairs, respectively, making the second setup more sensitive and a candidate for further development. In an experimental *T. brucei* mouse infection model, the optimized sdAbsR3-77H/sdAbR2-103HA sandwich ELISA assay detects its target antigen from day 6 post-infection onwards, which corresponds to the time point where the parasites also become detectable by microscopy. Interestingly, in subsequent days when parasitemia quickly drops below the microscopy detection limit, the sdAbsR3-77H/sdAbR2-103HA ELISA assay is still able to detect *Tbr*ENO. This is most likely due to parasites extravasating into tissues, which could include the adipose tissue and the brain, whereby actual blood parasitemia levels would become undetectable by microscopy. However, because *Tbr*ENO is known as part of the *T. brucei* secretome, it remains present in the host bloodstream as long as parasites are metabolically active in the body and thus accessible to the *Tbr*ENO-specific Abs of the test (30). Curative melarsoprol treatment leads to the rapid loss of the sdAbsR3-77H/sdAbR2-103HA ELISA signal, within 48 h after clearance of the infection, suggesting a target antigen half-life of approximately 30 h for *Tbr*ENO (see https://web.expasy.org). These findings add to the suitability of *Tbr*ENO as an infection biomarker and the further development of the sdAbsR3-77H/sdAbR2-103HA ELISA as a test of cure.

Furthermore, given the co-endemicity of other protozoan parasites within the African continent, such as *Leishmania* spp. and *Plasmodium* spp., we also performed a comparative alignment of enolase amino acid sequences from the species mentioned above using CLC Genomics Workbench 24. This revealed that *L. infantum* enolase shares ~80% sequence identity with *Tbr*ENO, while *Plasmodium* enolase displays ~60% sequence identity (see Fig. S1B). Hence, we performed an ELISA with plasma samples from mice infected with *P. chabaudi* and *L. infantum*, respectively, and compared the signal to that of plasma from *T. brucei*-infected mice. As shown in Fig. S2A, our results indicate strong specificity for the *T. brucei* enolase and plasma sample with no detectable signal observed for plasma from *P. chabaudi* and *L. infantum*. These data indicate that our assay might not suffer from cross-reactivity with other potentially co-endemic protozoan parasites. In addition, although *T. cruzi* is not endemic and thus not considered a co-infection problem, there is a high sequence homology of ~87% between *T. cruzi* and *T. brucei* enolases (Fig. S1B). Hence, we also tested if our assay might cross-react with this parasite using parasite lysates of both trypanosomes. Though the signals obtained for *T. brucei* using lysate are lower than those using plasma of infected animals, our results indicate no detectable signal observed for *T. cruzi* lysate (Fig. S2B), which suggests that our assay is a pan-specific test for detecting *T. brucei* parasites.

Finally, we also show that the assay has the potential to be suitable for testing human plasma samples of *T. b. rhodesiense* and *T. b. gambiense* HAT patients with a PPV that largely outperforms that of the currently available Ab test for gambiense HAT. Moreover, it allows the detection of human *T. b. rhodesiense* infections in both early and advanced stages, with increased signals obtained in the second stage. Given that (i) *T. b. rhodesiense* HAT is a zoonosis, (ii) there is no antibody test for *rhodesiense* HAT, and (iii) these infections generally present a high parasitemia (31, 32), the detection of ENO is hence an attractive solution. For *gambiense* HAT, the assay allows detection of chronic-stage infections, despite low parasitemia (2, 33). However, as reduced parasite loads can result in ENO concentrations that fall below the LoD of the sdAbsR3-77H/sdAbR2-103HA assay, negative results were obtained during the early stage of infection.

Despite the promising performance of the assay described in this study, several limitations should be considered. The first could be a reduced sensitivity for detecting low parasitemia, particularly in cases of West African HAT, where *T. b. gambiense* is endemic. In the chronic stage of gambiense HAT, parasitemia levels often fall below the assay’s detection threshold of 50,000 parasites/mL. This issue could be addressed by utilizing bivalent or multivalent sdAbs (25). While data derived from WHO Specimen Bank samples suggest that the assay performs well for moderate to high-parasitemia cases, further investigation is needed to evaluate its diagnostic accuracy in low-parasitemia cases. Additionally, the assay has not undergone extensive validation across a diverse range of clinical and field samples, including cerebrospinal fluid (CSF), which is needed for diagnosing late-stage HAT infections. Given the clearance of trypomastigotes from the bloodstream within 6–8 weeks in chronic gambiense HAT, a critical avenue for future investigation would be assessing the assay’s diagnostic utility on CSF samples from such cases.

Given its pan-specificity for detecting both HAT and AAT, the sdAbsR3-77H/sdAbR2-103HA ELISA could be a valuable surveillance tool for *T. brucei* infection epidemiology studies. Knowing that *T. brucei* species exhibit specific geographic distribution and host range, a single screening tool could play a role in a One Health approach across multiple regions. For (atypical) HAT, the sdAb-based assay may serve as an initial screening tool to support subsequent parasitological confirmation required for treatment decisions based on the disease stage of the infected individual. For AAT, the test results could lead to consistent treatment decisions with trypanocidal drugs across pathogens of the *T. brucei* species. Therefore, further evaluation and transformation of the sdAbsR3-77H/sdAbR2-103HA assay into a cost-effective lateral flow format merit consideration, as it delivers a pan-specific diagnostic test with properties desirable in remote and resource-poor settings.

## SUPPLEMENTAL MATERIAL

Figure S1 to S2 jcm.00561-25-s0001.docx Figure S1: Amino acid sequencing alignment of enolase from *Trypanosoma* spp., *Leishmania* spp., and *Plasmodium* spp. Figure S2: Specificity test using plasma from *T. brucei*, *P. chabaudi* and *L. infantum* infected mice, and lysate proteins from cultured *T. brucei* and *T. cruzi* parasites.

ASM does not own the copyrights to Supplemental Material that may be linked to, or accessed through, an article. The authors have granted ASM a non-exclusive, world-wide license to publish the Supplemental Material files. Please contact the corresponding author directly for reuse.

## References

[1] Balmer O, Beadell JS, Gibson W, Caccone A. 2011. Phylogeography and taxonomy of Trypanosoma brucei. PLoS Negl Trop Dis 5:e961. doi:10.1371/journal.pntd.0000961 21347445 PMC3035665

[2] Simarro P, Franco J, Diarra A, Jannin J. 2014. Epidemiology of human African trypanosomiasis. Clin Epidemiol 2014:257. doi:10.2147/CLEP.S39728 PMC4130665 25125985

[3] Hide G, Welburn SC, Tait A, Maudlin I. 1994. Epidemiological relationships of Trypanosoma brucei stocks from south east Uganda: evidence for different population structures in human infective and non-human infective isolates. Parasitology 109 (Pt 1):95–111. doi:10.1017/s0031182000077805 7914692

[4] MacLean L, Reiber H, Kennedy PGE, Sternberg JM. 2012. Stage progression and neurological symptoms in Trypanosoma brucei rhodesiense sleeping sickness: role of the CNS inflammatory response. PLoS Negl Trop Dis 6:e1857. doi:10.1371/journal.pntd.0001857 23145191 PMC3493381

[5] Joshi PP, Shegokar VR, Powar RM, Herder S, Katti R, Salkar HR, Dani VS, Bhargava A, Jannin J, Truc P. 2005. Human trypanosomiasis caused by Trypanosoma evansi in India: the first case report. Am J Trop Med Hyg 73:491–495. doi:10.4269/ajtmh.2005.73.491 16172469

[6] Vanhollebeke B, Truc P, Poelvoorde P, Pays A, Joshi PP, Katti R, Jannin JG, Pays E. 2006. Human Trypanosoma evansi infection linked to a lack of apolipoprotein L-I. N Engl J Med 355:2752–2756. doi:10.1056/NEJMoa063265 17192540

[7] Chitanga S, Marcotty T, Namangala B, Van den Bossche P, Van Den Abbeele J, Delespaux V. 2011. High prevalence of drug resistance in animal trypanosomes without a history of drug exposure. PLoS Negl Trop Dis 5:e1454. doi:10.1371/journal.pntd.0001454 22206039 PMC3243716

[8] Tchamdja E, Kulo AE, Vitouley HS, Batawui K, Bankolé AA, Adomefa K, Cecchi G, Hoppenheit A, Clausen PH, De Deken R, Van Den Abbeele J, Marcotty T, Delespaux V. 2017. Cattle breeding, trypanosomosis prevalence and drug resistance in Northern Togo. Vet Parasitol 236:86–92. doi:10.1016/j.vetpar.2017.02.008 28288771

[9] Odiit M, Coleman PG, Liu W ‐C., McDermott JJ, Fèvre EM, Welburn SC, Woolhouse MEJ. 2005. Quantifying the level of under‐detection of Trypanosoma brucei rhodesiense sleeping sickness cases. Tropical Med Int Health 10:840–849. doi:10.1111/j.1365-3156.2005.01470.x 16135190

[10] Eberhardt AT, Monje LD, Zurvera DA, Beldomenico PM. 2014. Detection of Trypanosoma evansi infection in wild capybaras from Argentina using smear microscopy and real-time PCR assays. Vet Parasitol 202:226–233. doi:10.1016/j.vetpar.2014.02.043 24636712

[11] Desquesnes M, Dávila AMR. 2002. Applications of PCR-based tools for detection and identification of animal trypanosomes: a review and perspectives. Vet Parasitol 109:213–231. doi:10.1016/s0304-4017(02)00270-4 12423934

[12] Kuboki N, Inoue N, Sakurai T, Di Cello F, Grab DJ, Suzuki H, Sugimoto C, Igarashi I. 2003. Loop-mediated isothermal amplification for detection of African trypanosomes. J Clin Microbiol 41:5517–5524. doi:10.1128/JCM.41.12.5517-5524.2003 14662933 PMC308967

[13] Li Z, Pinto Torres JE, Goossens J, Stijlemans B, Sterckx YG-J, Magez S. 2020. Development of a recombinase polymerase amplification lateral flow assay for the detection of active Trypanosoma evansi infections. PLoS Negl Trop Dis 14:e0008044. doi:10.1371/journal.pntd.0008044 32069278 PMC7048301

[14] Kim J, Álvarez-Rodríguez A, Li Z, Radwanska M, Magez S. 2023. Recent progress in the detection of Surra, a neglected disease caused by Trypanosoma evansi with a one health impact in large parts of the tropic and sub-tropic world. Microorganisms 12:44. doi:10.3390/microorganisms12010044 38257871 PMC10819111

[15] Espino AM, Millán JC, Finlay CM. 1992. Detection of antibodies and circulating excretory-secretory antigens for assessing cure in patients with fascioliasis. Trans R Soc Trop Med Hyg 86:649. doi:10.1016/0035-9203(92)90174-b 1287933

[16] Guobadia EE, Fagbemi BO. 1995. Time-course analysis of antibody response by EITB and ELISA before and after chemotherapy in sheep infected with Fasciola gigantica. Vet Parasitol 58:247–253. doi:10.1016/0304-4017(94)00721-n 7571329

[17] Kayang BB, Bosompem KM, Assoku RK, Awumbila B. 1997. Detection of Trypanosoma brucei, T. congolense and T. vivax infections in cattle, sheep and goats using latex agglutination. Int J Parasitol 27:83–87. doi:10.1016/s0020-7519(96)00160-9 9076533

[18] Nantulya VM, Doua F, Molisho S. 1992. Diagnosis of Trypanosoma brucei gambiense sleeping sickness using an antigen detection enzyme-linked immunosorbent assay. Trans R Soc Trop Med Hyg 86:42–45. doi:10.1016/0035-9203(92)90435-f 1566302

[19] Rebeski DE, Winger EM, Van Rooij EM, Schöchl R, Schuller W, Dwinger RH, Crowther JR, Wright P. 1999. Pitfalls in the application of enzyme-linked immunoassays for the detection of circulating trypanosomal antigens in serum samples. Parasitol Res 85:550–556. doi:10.1007/s004360050594 10382604

[20] Kashiwazaki Y, Thammasart S. 1998. Effect of anti-immunoglobulin antibodies produced in cattle infected with Trypanosoma evansi on antigen detection ELISA. Int J Parasitol 28:1353–1360. doi:10.1016/s0020-7519(98)00123-4 9770620

[21] Zhu M, Gong X, Hu Y, Ou W, Wan Y. 2014. Streptavidin-biotin-based directional double Nanobody sandwich ELISA for clinical rapid and sensitive detection of influenza H5N1. J Transl Med 12:352. doi:10.1186/s12967-014-0352-5 25526777 PMC4274719

[22] Hamers-Casterman C, Atarhouch T, Muyldermans S, Robinson G, Hamers C, Songa EB, Bendahman N, Hamers R. 1993. Naturally occurring antibodies devoid of light chains. Nature 363:446–448. doi:10.1038/363446a0 8502296

[23] Dooley H, Flajnik MF, Porter AJ. 2003. Selection and characterization of naturally occurring single-domain (IgNAR) antibody fragments from immunized sharks by phage display. Mol Immunol 40:25–33. doi:10.1016/s0161-5890(03)00084-1 12909128

[24] Muyldermans S, Atarhouch T, Saldanha J, Barbosa J, Hamers R. 1994. Sequence and structure of V_H_ domain from naturally occurring camel heavy chain immunoglobulins lacking light chains. Protein Eng Des Sel 7:1129–1135. doi:10.1093/protein/7.9.1129 7831284

[25] Pinto Torres JE, Goossens J, Ding J, Li Z, Lu S, Vertommen D, Naniima P, Chen R, Muyldermans S, Sterckx YG-J, Magez S. 2018. Development of a Nanobody-based lateral flow assay to detect active Trypanosoma congolense infections. Sci Rep 8:9019. doi:10.1038/s41598-018-26732-7 29899344 PMC5998082

[26] Li Z, Pinto Torres JE, Goossens J, Vertommen D, Caljon G, Sterckx YG-J, Magez S. 2020. An unbiased immunization strategy results in the identification of enolase as a potential marker for nanobody-based detection of Trypanosoma evansi. Vaccines (Basel) 8:8. doi:10.3390/vaccines8030415 PMC7565430 32722150

[27] Lanham SM, Godfrey DG. 1970. Isolation of salivarian trypanosomes from man and other mammals using DEAE-cellulose. Exp Parasitol 28:521–534. doi:10.1016/0014-4894(70)90120-7 4993889

[28] De Vlaminck K, Van Hove H, Kancheva D, Scheyltjens I, Pombo Antunes AR, Bastos J, Vara-Perez M, Ali L, Mampay M, Deneyer L, Miranda JF, Cai R, Bouwens L, De Bundel D, Caljon G, Stijlemans B, Massie A, Van Ginderachter JA, Vandenbroucke RE, Movahedi K. 2022. Differential plasticity and fate of brain-resident and recruited macrophages during the onset and resolution of neuroinflammation. Immunity 55:2085–2102. doi:10.1016/j.immuni.2022.09.005 36228615

[29] Odongo S, Sterckx YGJ, Stijlemans B, Pillay D, Baltz T, Muyldermans S, Magez S. 2016. An anti-proteome nanobody library approach yields a specific immunoassay for Trypanosoma congolense diagnosis targeting glycosomal aldolase. PLoS Negl Trop Dis 10:e0004420. doi:10.1371/journal.pntd.0004420 26835967 PMC4737498

[30] Szempruch AJ, Sykes SE, Kieft R, Dennison L, Becker AC, Gartrell A, Martin WJ, Nakayasu ES, Almeida IC, Hajduk SL, Harrington JM. 2016. Extracellular vesicles from Trypanosoma brucei mediate virulence factor transfer and cause host anemia. Cell 164:246–257. doi:10.1016/j.cell.2015.11.051 26771494 PMC4715261

[31] Odiit M, Kansiime F, Enyaru JC. 1997. Duration of symptoms and case fatality of sleeping sickness caused by Trypanosoma brucei rhodesiense in Tororo, Uganda. East Afr Med J 74:792–795. 9557424

[32] Checchi F, Filipe JAN, Barrett MP, Chandramohan D. 2008. The natural progression of Gambiense sleeping sickness: what is the evidence? PLoS Negl Trop Dis 2:e303. doi:10.1371/journal.pntd.0000303 19104656 PMC2602732

[33] Checchi F, Filipe JAN, Haydon DT, Chandramohan D, Chappuis F. 2008. Estimates of the duration of the early and late stage of gambiense sleeping sickness. BMC Infect Dis 8:1–10. doi:10.1186/1471-2334-8-16 18261232 PMC2259357

