# Supplementary material for: Development of a camelid single-domain antibody-based antigen detection assay for the pan-specific diagnosis of active human and animal Trypanosoma brucei infections
Source: J Clin Microbiol. 2025 Oct 30;63(12):e00561-25. doi: 10.1128/jcm.00561-25 (PMC12710306; doi:10.1128/jcm.00561-25)
Supplement: Figure S1 to S2 — Figure S1: Amino acid sequencing alignment of enolase from Trypanosoma spp., Leishmania spp., and Plasmodium spp. Figure S2: Specificity test using plasma from T. brucei, P. chabaudi and L. infantum infected mice, and lysate proteins from cultured T. brucei and T. cruzi parasites. [file jcm.00561-25-s0001.docx]

**Supplementary**


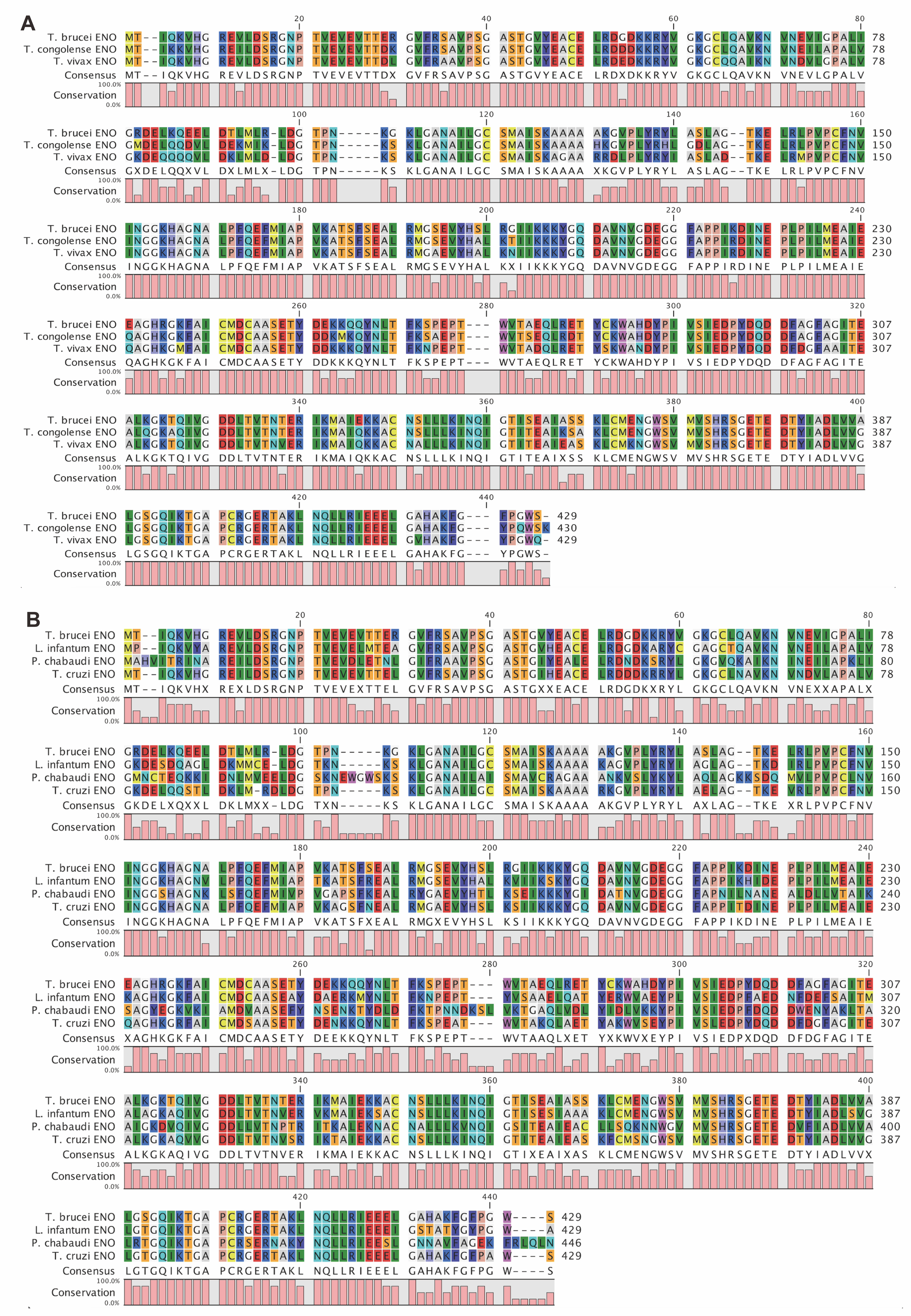


**Figure S1. Amino acid sequencing alignment of enolase from *Trypanosoma* spp., *Leishmania* spp., and *Plasmodium* spp.** The sequencing alignment was performed with CLC Genomics Workbench 24. A, Amino acid sequencing alignment of enolase from *T. brucei*, *T. congolense,* and *T. vivax*. The sequences were retrieved from TriTrypDB, *T. brucei* ENO (Transcrip ID: Tb927.10.2890), *T. congolense* ENO (Transcrip ID: TcIL3000.A.H_000739900.1), *T. vivax* ENO (Transcrip ID: TvY486_1002910). B, Amino acid sequencing alignment of enolase from *T. brucei*, *L. infantum, P. chabaudi* and *T. cruzi.* The enolase sequences of *T. brucei*, *L. infantum,* and *T. cruzi* were retrieved from TriTrypDB, *T. brucei* ENO (Transcrip ID: Tb927.10.2890), *L. infantum* ENO (Transcrip ID: LINF_140018000-T1) and *T. cruzi* ENO (Transcrip ID: BCY84_15382_t1 GenBank: KAF8293506.1)*,* and *P. chabaudi* ENO (Transcrip ID: PCHAS_1215000.1) was retrieved from PlasmoDB.


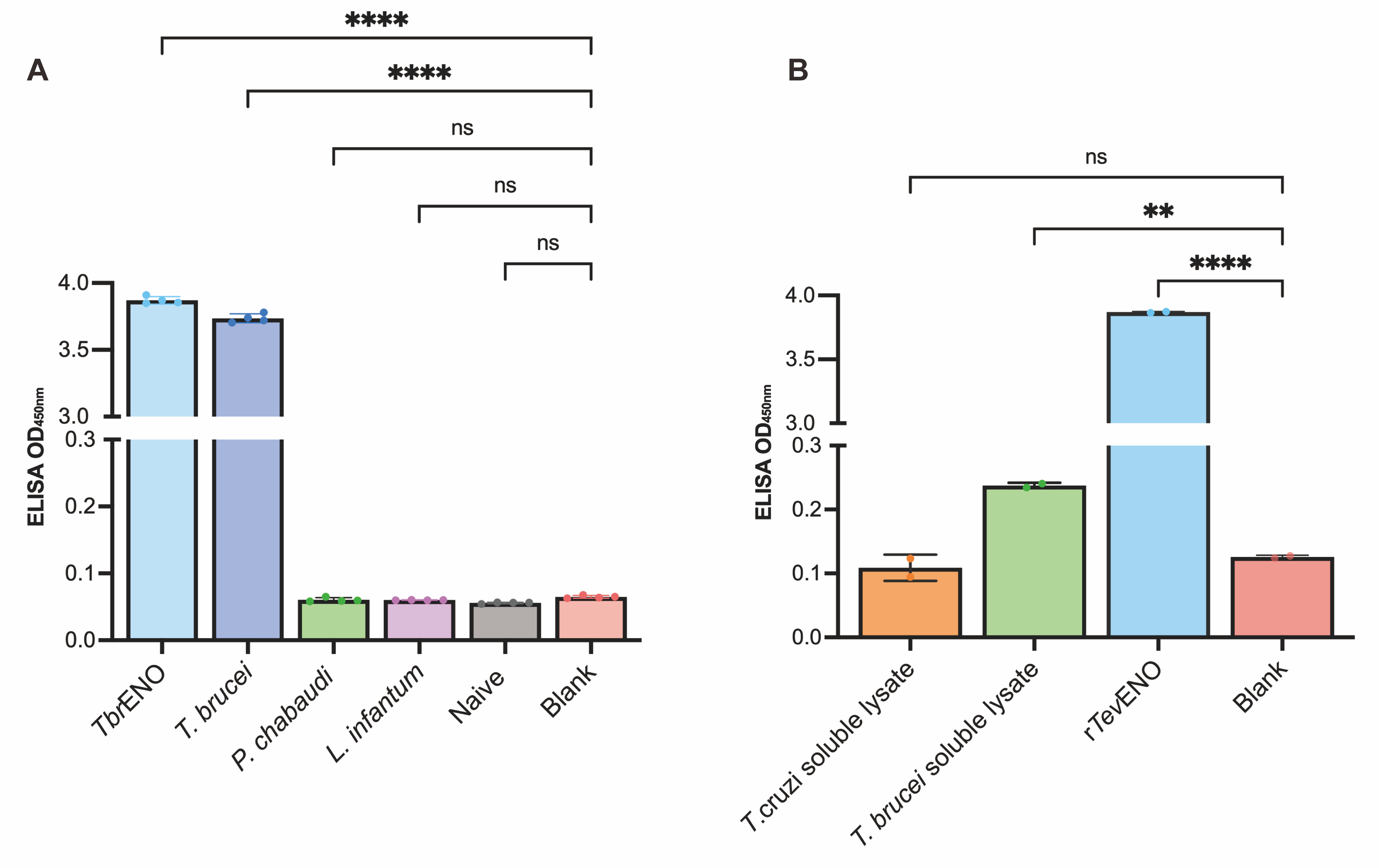


**Figure S2. Specificity test using plasma from *T. brucei*, *P. chabaudi* and *L. infantum* infected mice, and lysate proteins from cultured *T. brucei* and *T. cruzi* parasites.** A. Specificity test using plasma samples from mice infected with *T. brucei*, *P. chabaudi* and *L. infantum*. For each group, four individuals were used as replicates. Plasma from *T. brucei* infected mice and recombinant *Tbr*ENO were used as positive controls, while plasma from naïve mice served as a negative control. A one-way ANOVA (Kruskal-Wallis test with multiple comparisons) was performed, comparing each sample to the blank. Results are representative of two independent experiments and presented as mean ± SD (****: p-values ≤0.0001, ns: not significant). B. Specificity test using soluble lysate proteins derived from *in vitro* cultured *T. brucei* and *T. cruzi* parasites. A one-way ANOVA (Kruskal-Wallis test with multiple comparisons) was performed, whereby each sample was compared to the blank. Results are representative of 2 independent experiments (*n* = 2) and presented as mean ± SD (**: p-values ≤0.01, ns: not significant).
